# Post-Embryonic Phase Transitions Mediated by Polycomb Repressive Complexes in Plants

**DOI:** 10.3390/ijms22147533

**Published:** 2021-07-14

**Authors:** Valerie Hinsch, Samuel Adkins, Darren Manuela, Mingli Xu

**Affiliations:** Department of Biological Sciences, University of South Carolina, Columbia, SC 29208, USA; vhinsch@email.sc.edu (V.H.); swadkins@email.sc.edu (S.A.); manuelad@email.sc.edu (D.M.)

**Keywords:** PRC1, PRC2, H3K27me3, chromatin, embryo-to-seedling transition, vegetative phase transition, floral induction

## Abstract

Correct timing of developmental phase transitions is critical for the survival and fitness of plants. Developmental phase transitions in plants are partially promoted by controlling relevant genes into active or repressive status. Polycomb Repressive Complex1 (PRC1) and PRC2, originally identified in Drosophila, are essential in initiating and/or maintaining genes in repressive status to mediate developmental phase transitions. Our review summarizes mechanisms in which the embryo-to-seedling transition, the juvenile-to-adult transition, and vegetative-to-reproductive transition in plants are mediated by PRC1 and PRC2, and suggests that PRC1 could act either before or after PRC2, or that they could function independently of each other. Details of the exact components of PRC1 and PRC2 in each developmental phase transitions and how they are recruited or removed will need to be addressed in the future.

## 1. Introduction

Plants develop from embryos to seedlings when they are exposed to germination-inducing factors, followed afterward by juvenile and adult vegetative development. Upon floral induction, plants transition from vegetative to reproductive development. The correct timing of these developmental phase transitions in plants is essential to the fitness and reproductive success of the plant and is therefore tightly controlled by both endogenous and environmental cues [1]. These cues affect the expression of key developmental transition genes spatially and/or temporally to facilitate and/or maintain developmental phase transitions. The key developmental phase transition genes are regulated by transcription factors and epigenetic factors, which together fine tune these developmental phase transitions. The DNA of these key genes is first packaged into nucleosomes, which are octameric complexes that consist of two molecules of H3, H4, H2A, and H2B, with roughly 1.7 turns of DNA, equaling approximately 147 base pairs twisted around the octamer and locked by one H1 [2]. The N-terminal tail of histones extends out of the nucleosome package and can be modified to form “histone codes” that can then be read by other proteins to mediate activation or repression of the genes [3,4].

Modifications made to histone H3 are important in regulating gene expression. The degree and location of lysine methylation on H3 can either promote or repress transcription, termed “active” or “repressive” markers, respectively. The trimethylation of lysine 4 (H3K4me3) and 36 (H3K36me3), are typically “active” markers whereas H3K9me3 and H3K27me3 are typically “repressive” markers [5]. H3K27me3 is strongly associated with gene repression, and is considered essential for gene silencing due to the conserved pathways for depositing H3K27me3 in both animals and plants [5]. Genome-wide analysis of H3K27me3 in *Arabidopsis* (*Arabidopsis thaliana*) revealed more than 4400 genes actively marked by H3K27me3 in seedlings, and 9006 genes marked by H3K27me3 in shoot apical meristem and leaves [6,7]. The methylation of H3K27 is carried out by histone methyltransferases (HMTases), while demethylases remove the methylation [5]. In both animals and plants, Polycomb group (PcG) proteins are primarily responsible for gene silencing via the H3K27 methylation. The PcG proteins were first described in *Drosophila* (*Drosophila melanogaster*) by E.B. Lewis, where they were found to have a repressive function on the regulation of growth and body segmentation [8]. After this, the Polycomb repressive complex 1 (PRC1) was purified and found to contain Polycomb (Pc), Posterior Sex Combs (Psc), Polyhomeotic (Ph), Sex Combs on Midleg (Sce), and other proteins [9]. This was followed by the discovery of the PRC2 by identifying its Enhancer of Zeste (EZ) component [10]. In *Drosophila*, the PRC2 contains four core constituents: Enhancer of Zeste (E(z)), a histone methyltransferase (HMTase); Extra Sex Combs (ESC), a WD-40 domain protein; Suppressor of Zeste 12 (Su(z)12), a zinc finger protein; and a 55 kDa nucleosome remodeling factor (Nurf55 or p55). Both PRC1 and PRC2 are involved in H3K27me3 mediated gene silencing but act differently. PRC2 has HMTase activity that mediates the methylation of H3K27. PRC1 does not have HMTase, but it can be recruited by H3K27me3 to induce monoubiquitination of H2A and heterochromatin status. Studies also show that PRC1 may act earlier than PRC2 [11]. Homologs of PRC1 and PRC2 have been found in plants to be involved in a wide range of developmental processes which include embryo development after fertilization, seed maturation, vegetative phase change, flowering, and stress responses [12,13]. Here, we will focus on the roles of PRC1 and PRC2 in regulating post-embryonic developmental phase transitions, including the embryo-to-seedling transition, juvenile-to-adult vegetative phase transition, and vegetative-to-reproductive phase transition.

## 2. PRC1 and PRC2 in Plants

### 2.1. PRC2 in Plants

Though PRC1 was discovered earlier than PRC2 [8,9,10], PRC2 has been found to typically act ahead of PRC1 in mediating gene silencing [9]. In plants, PRC2 components and their roles were discovered prior to PRC1 [14,15,16]. Accordingly, we will discuss the roles of PRC2 in mediating plant phase transitions first. In *Arabidopsis*, HMTases SWINGER (SWN), CURLY LEAF (CLF) and MEDEA (MEA) are homologous to the *Drosophila* E(z). Zinc finger proteins EMBRYONIC FLOWER2 (EMF2), FERTILIZATION INDEPENDENT SEEDS2 (FIS2), and VERNALIZATION2 (VRN2) are homologous to the *Drosophila* Su(z)12. WD-40 protein FERTILIZATION INDEPENDENT ENDOSPERM (FIE) is homologous to the *Drosophila* Esc, and MULTIPLE SUPPRESSOR OF IRA1-5 (MSI1-5) are homologous to p55/Nurf55 (Table 1). Among the *Arabidopsis* core PRC2 components, *FIE* is expressed in all cell types in both the nucleus and cytoplasm, and has no alternatives [17,18,19]. The other components of PRC2 are more cell specific and form different PRC2 complexes to mediate different developmental phase transitions. The PRC2 components combine in different ways to form three distinct PRC2 complexes with overlapping function, categorized by the homologous Su(z)12 component they receive. The EMF2-complex has been found to suppress precocious reproductive development after germination [20]. The VRN2-complex serves as a regulator of vernalization, in which extended exposure to cold temperatures mediates silencing of the MADS-box gene *FLOWERING LOCUS C* (*FLC*) [21]. The FIS2-complex is responsible for the repression of seed development without fertilization, and any mutations or loss of subunits within this complex resulted in seed abortion [22]. PRC2 complexes are critical for cellular differentiation and proliferation in plants by regulating a host of genes with a wide array of functions [23].

### 2.2. PRC1 in Plants

In animals, stable repression by H3K27me3 requires the activities of PRC1, which is recruited by H3K27me3 and catalyzes the monoubiquitination of histone H2A at lysine 119 (H2AK119ub1) to induce formation of heterochromation and maintaining transcriptional silencing [24]. The existence of plant PRC1 had been doubted for many years until two groups reported that LIKE-HETEROCHROMATIN PROTEIN 1/TERMINAL FLOWER 2 (LHP1/TFL2), shares high similarities to the HP1 of metazoans. LHP1 binds to H3K27me3 in vitro and colocalizes with H3K27me3 in *Arabidopsis*, suggesting that LHP1/TFL2 is a homolog of Pc in plants [25,26]. Homologs of Sce, *Arabidopsis thaliana* REALLY INTERESTING NEW GENE 1A (AtRING1A) and AtRING1B, and homologs of Psc, *Arabidopsis thaliana* B LYMPHOMA MO-MLV INSERTION REGION 1A (AtBMI1A/AtBMIB/AtBMI1C) were discovered afterwards, while a homolog of Ph have not yet been reported [11,14,27,28,29,30] (). Among those PRC1 components, AtBMI1 proteins display E3 H2A monoubiquitin ligase activity [14,30]. Disrupting LHP1 resulted in loss of H3K27me3 binding and derepressing of silenced genes [31]. EMBRYONIC FLOWER 1 (EMF1) and VERNALIZATION 1 (VRN1) are two plant specific PRC1 components. EMF1 has an ATP/GTP binding motif (P-loop) and LXXLL motif [32] and physically interacts with AtRING1A/B and AtBMI1A/B/C to assist with the ubiquitination of H2A [14,15,30]. AtBMI1A/B/C also mediate the ubiquitination of H2A variant H2A.Z [33]. The PRC1 components are widely present in gymnosperms and angiosperms, indicating conserved roles of PRC1 in higher plants [34]. Though PRC1 was originally identified as a suppressor for embryonic traits, genome-wide analysis showed that PRC1 components AtRING1A/B, AtBMI1A/B/C, EMF1 and LHP1 act not only as repressors but also as activators, and they act in all plant developmental stages [35]. 

### 2.3. PRC1 and PRC2 Interactions in Plants

**Figure 1:**
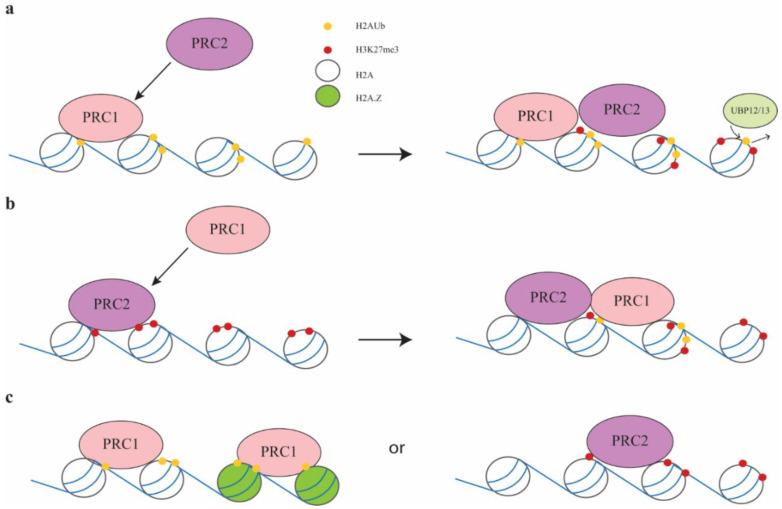
Model of PRC1 and PRC2 interactions. (**a**) PRC1 acts first and induces ubiquitination of H2A. PRC2 is recruited after PRC1, and H3K27me3 is deposited. H2Aub is then removed by UBP12/13 to maintain H3K27me3. (**b**) PRC2 acts first and deposits H3K27me3; PRC1 is recruited by H3K27me3; and H2A is ubiquitinated afterwards. (**c**) PRC1 and PRC2 act independently of each other. Both H2A and H2A.Z could be ubiquitinated.

The intricacies of the interaction between PRC1 and PRC2 are still under review. The MSI1 protein binds to LHP1 and EMF1, indicating the connection between PRC1 and PRC2 [15,36]. It has been shown in animals that PRC1 is recruited by H3K27me3 [10,37] (Figure 1b). However, this model was challenged by Blackledge et al., who demonstrated that knocking down PRC1 in mouse stem cells not only resulted in loss of H2AK119ub, but also reduced PRC2 binding [38], suggesting that PRC1 may act before PRC2. This result has also been observed in *Arabidopsis*, where mutations in *EMF1* and *ATBMI1A/1B/1CAtbmia/b/c* resulted in reduced levels of H2Aub and H3K27me3 at some PRC2 targets [11,15] (Figure 1a). 

Several studies have indicated that PRC1 may act independently of PRC2. Levels of H2Aub are almost absent in *atbmi1a/b* mutants, but unchanged in *clf/swn* mutants, suggesting that PRC2 may not be necessary for PRC1 binding [11]. Genome wide profiling of H2AK121ub (monoubiquitination of lysine 121 on H2A) and H3K27me3 marks in 7-day-old seedlings showed that 14,088 genes are marked by H2AK121ub, and among them 4979 genes are co-marked by H3K27me3. The remaining 9109 genes are marked by H2AK121ub only, suggesting that a large number of genes are targeted by PRC1 but not PRC2 [39]. Further analysis of H2AK121ub and H3K27me3 in *atbmi1a/b/c*, *clf/swn*, and *lhp1* mutants showed that levels of H2AK121ub are significantly reduced in *atbmi1a/b/c* triple mutant [39]. However, most of the genes marked only by H2AK121ub did not change in *clf/swn* double mutant [39], suggesting that PRC2 activity is not required for establishing H2AK121ub at these targets. Nevertheless, a large portion of the H2AK121ub and H3K27me3 co-marked genes have reduced levels of H2AK121ub and H3K27me3 in *atbmi1a/b/c* [39]. In addition, the analysis of monoubiquitination of H2A variant H2A.Z showed that H2A.Z could also be monoubiquitinated by AtBMI1A/B/C, but this modification and subsequent transcriptional repression does not require PRC2 activity, supporting the hypothesis that PRC1 may act independently of PRC2 [33] (Figure 1c). In plants, the ubiquitination at H2A could be removed by two functionally redundant factors Ubiquitin-Specific Proteases 12 and 13 (UBP12 and UBP13) [40,41]. Recent genome-wide studies revealed that PRC1 deposits H2Aub1 and initiates transcriptional repression, after which PRC2 is recruited, and finally UBP12/13 removes H2Aub1 for stable silencing (Figure 1a). Some genes are expressed at low levels and do not require PRC1 to initiate the repression, so only PRC2 is recruited to these targets for stable silencing [40] (Figure 1c). Together, these studies indicate that PRC2 and PRC1 act independently at some targets, while both PRC1 and PRC2 are required for H3K27me3 at other targets. 

## 3. Post-Embryonic Developmental Transitions Mediated by PRC2 and PRC1

### 3.1. Embryo-to-Seedling Transition Mediated by PRC2 and PRC1

In *Drosophila*, loss of PRC2 function substantially alters oocyte cell fate transition and body patterning [42,43]. In *Arabidopsis*, the *fie* single mutants, *clf/swn* double mutants and *emf2/vrn2* double mutants grow normally and initiate body patterns after germination, but later develop callus-like structures rather than leaf-like structures. Consistent with this, the amount of H3K27me3 is significantly reduced in these mutants [7,16,44,45], suggesting that the deposition of H3K27me3 by PRC2 components CLF, SWN, EMF2, VRN2, and FIE is required for cell differentiation and organogenesis during the embryo-to-seedling phase transition. MSI1 is likely to be the functional p55 homolog during this transition, as the strong allele of *msi1* is embryonic lethal [46,47]. Genome-wide analysis shows that genes encoding protein for auxin biosynthesis or auxin signaling, genes encoding a family of MADS transcription factors, genes encoding a family of LATE EMBRYOGENESIS ABUNDANT proteins, and genes encoding a family of oil surface proteins are all marked by H3K27me3 and they were upregulated in *fie* mutant, suggesting that they may be involved in promoting embryo-to-seedling transition [44]. 

Genetic analysis of PRC1 components shows that members of the *AtRING1A/B* and *AtBMI1A/B/C* are redundant, and the strong alleles of *atring1a/b* and *atbmi1a/b/c* mutants have similar phenotypes to the *clf/swn* mutants, and also develop callus-like structures rather than leaves after germination [14,27,30]. AtRING1A/B and AtBMI1A/B/C proteins have a conserved RING finger domain, and molecular analysis demonstrates that they can monoubiquitinate Lys121 of H2A at embryonic genes *FUSCA3* (*FUS3*), *LEAFY COTYLENDON1* (*LEC1*), *LEC2*, and *ABSCISIC ACID INSENSITIVE3* (*ABI3*); meristematic genes *WUSCHEL* (*WUS*) and *WUSCHEL RELATED HOMEOBOX5* (*WOX5*); and Class I homeobox KNOX genes *SHOOTMERISTEMLESS* (*STM*), *KNOTTED LIKE FROM ARABIDOPSIS THALIANA* (*KNAT1*), *KNAT2* and *KNAT6* [11,14,28,30,48]. Consistent with this, these genes are derepressed in *atring1a/b*, and *atbmi1a/b/c*. Levels of H3K27me3 in *atbmi1a/b/c* are strongly reduced at *LEC1*, *ABI3* and *FUS3*, but not at *STM* nor *WUS* [11]. These results suggest that the PRC1-dependent activity of PRC2 may be target-specific. 

**Figure 2:**
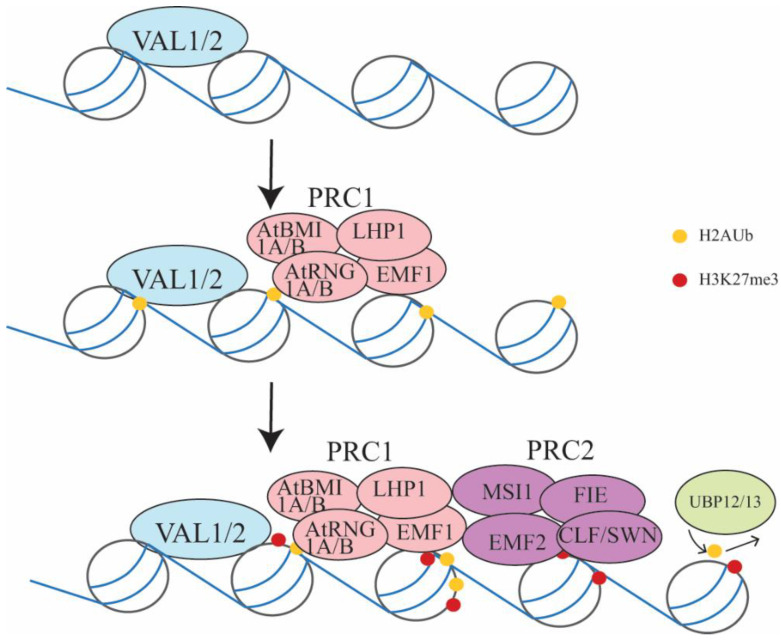
Model of PRC1 and PRC2 during embryo-to-seedling transition. Recruitment of VAL1/2 to the chromatin of LEC1/LEC2/ABI3/FUS3 or other targets to mediate binding of PRC1 via interaction with the AtBMI1A/B complex. PRC1 ubiquitinates H2A and recruits PRC2 via binding of MSI1 (a PRC2 component) to EMF1/LHP1(PRC1 component), leading to increased levels of H3K27me3 at LEC1/LEC2/ABI3/FUS3 and other genes. UBP12/13 removes ubiquitin from target sites to allow binding of PRC2 and stable silencing.

Several proteins have been found to assist with PRC1 function during seedling development. Pull-down assays showed that the B3 transcription factors VP1/ABI3-LIKE 1/2 (VAL1/2) physically interact with AtBMI1A/B [11]. Genetic analysis showed that *val1/2* double mutant is arrested in development after germination, and embryonic development related genes *LEC1*/*LEC2*/*ABI3* are upregulated in *val1/2*, resembling *atbmi1a/b* mutants [11]. VAL1/2 also bind to the RY motif of LEC2/ABI3 and mediate the recruitment of PRC1 [11,35] (Figure 2). Additionally, ALFIN1-like proteins (ALs) and AtZUOTIN-RELATED FACTOR1 (AtZRF1) proteins were found to interact with PRC1 components for H2Aub1 in regulating seed germination. The RAWUL domain of AtRING1A and AtBMI1B, which is conserved in animals and plants, interacts with the N-terminal region of the AL6 protein [49]. a*l6/7* mutant seeds are delayed in germination under osmotic stress, which could be resulted from the derepression of *ABI3*, *DELAY OF GERMINATION 1* (*DOG1*), *CRUCIFERIN1* (*CRU,*), *CRU3*, and *CHOTTO1* (*CHO1*). H3K27me3 levels are reduced in *al6 al7* double mutants, but it is not clear if H2AK121ub is reduced in *al* mutants. Due to the binding of AL proteins to H3K4me3, it has been proposed that AL binds to H3K4me3 and recruits PRC1 and then PRC2 to catalyze the methylation to H3K27 [49,50]. The AtARF1 in *Arabidopsis* are found to play a role in seed germination, similar to AtRING1A/B and AtBMI1A/B [51]. The levels of H3K27me3 and H2Aub1 are reduced at *ABI3* and *CRU3* gene bodies, suggesting the role of AtZRF proteins as PRC1/PRC2 co-factors that assist with PRC1/PRC2 binding [51]. 

It is worth noting that mutations in a CHD3 chromatin-remodeling factor *PICKLE* (*PKL*) result in reduced levels of H3K27me3 and derepression of embryonic genes *LEC2* and *FUS3* among other genes [52,53,54]. PKL functions antagonistically to CLF in maintaining root meristem activity [55,56]. There is evidence that PKL proteins directly interact with the promoter regions of H3K27me3-enriched genes in shoots [52], and PKL is reported to regulate H3K27me3 abundancy at embryonic genes *LEC2* and FUS3 [52,53]. However, it is still unknown how PKL interacts with PRC2 or PRC1 to facilitates the deposition of H3K27me3 at embryonic genes during germination. 

### 3.2. Juvenile-to-Adult Vegetative Phase Transition Mediated by PRC2 and PRC1

After the establishment of seedlings from embryos, plants enter the vegetative phase of development. Lateral organs (leaves and axillary buds) produced from the shoot apical meristem (SAM) during the vegetative phase are morphologically different and their heteroblastic traits change over time. The heteroblastic traits are species-specific, but the changes within a species are coordinated and predictable. Accordingly, the vegetative phase is divided into a juvenile phase and an adult phase [57,58,59]. The transition between these phases is termed “vegetative phase change” and is mediated by a decrease in the expression of two related microRNAs, miR156 and miR157. A group of SQUAMOSA PROMOTER BINDING PROTEIN-LIKE (SPL) transcription factors are targeted by miR156/157 to induce a wide range of changes, including juvenile-to-adult vegetative phase change, lateral root development, adventitious root development, competence to flowering, and response to stressors, including low temperature, senescence, pathogens and herbivores [60,61,62,63,64,65,66,67,68]. The roles of the miR156-SPL module are conserved in annual and perennial plants [66,67,69,70]. Studies in *Arabidopsis* reveal that miR156 and its targeted SPLs are regulated by sugar [71,72], transcription factors [73,74], and epigenetic factors [75,76,77,78,79]. 

**Figure 3:**
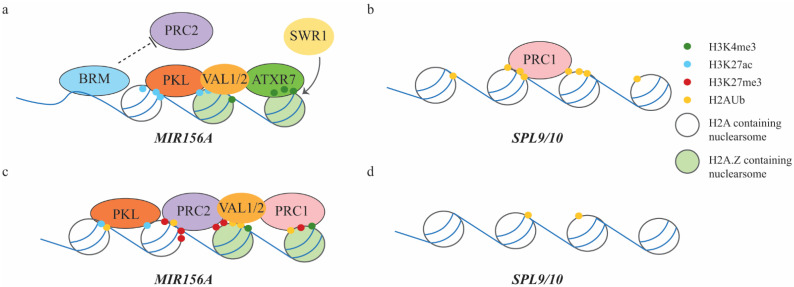
Model of PRC1 and PRC2 action during juvenile-to-adult vegetative phase change. (**a**,**b**) PRC1 and PRC2 during juvenile phase. (**a**) BRM prevents the formation of nucleosomes near the transition start site (TSS) of *MIR156A* and possibly prevents binding of PRC2 to *MIR156A*. PKL binds to *MIR156A* during juvenile phase and is associated with modulating H3K27ac levels. SWR1 mediates the exchange of H2A to H2A.Z, which works in concert with ATXR7 to deposit H3K4me3. (**b**) SPL9 and SPL10 are marked by H2Aub that deposited by PRC1 to prevent precocious vegetative phase change. (**c**,**d**) PRC1 and PRC2 during adult phase. (**c**) PKL facilitates nucleosome formation near the TSS and PRC1 and PRC2 are recruited to *MIR156A* to increase the abundance of H3K27me3 and H2AUb, while the abundance of H3K27ac and H3K4me3 are decreased. VAL1/2 bind to *MIR156A* to recruit PRC1 and PRC2. (**d**) PRC1 leaves SPL9/10 to allow for their activation.

In *Arabidopsis*, miR156 is encoded by eight genes, *MIR156A*-*MIR156H*. Genetic analysis indicates that *MIR156A/MIR156C* are the major sources for mature miR156 [62,72]. Mutant screening led to the discovery that PKL, a CHD3 chromatin remodeling factor mentioned previously, regulates vegetative phase change. Nucleosomes are physical barriers for RNA polymerase II, so chromatin remodeling factors likely affect gene transcription by modulating nucleosome occupancy near the transcription start site [80]. PKL promotes the +1 nucleosome occupancy at *MIR156A/MIR156C* [75]. During vegetative phase change, primary transcripts of *MIR156A/MIR156C* are temporally downregulated, correlating with the temporal decrease in H3K27ac, an active marker, and temporal increase in H3K27me3, a repressive marker [52,53]. It has been found that PKL interacts synergistically with PRC2 component SWN to modulate the levels of H3K27ac and H3K27me3 at *MIR156A/MIR156C* [75]. In contrast to PKL, another chromatin remodeling factor and component of the SWI2/SNF2 chromatin remodeling complex, ATPase BRAMAH (BRM) acts antagonistically to SWN, preventing the +1 nucleosome accumulation and allowing active transcription of *MIR156A* [77]. Therefore, PKL, BRM, and PRC2 are involved in mediating temporal deposition of H3K27me3 at *MIR156A/MIR156C* (Figure 3a,c). BRM prevents the binding of PRC2 at a flowering repressor *SHORT VEGETATIVE PHASE* [81], and it is likely that BRM may prevent the binding of PRC2 at *MIR156A*. However, it is still unknown how PKL mediate the activities of PRC2. 

Levels of H3K27me3 are anti-correlated with the levels of H3K4me3, an active marker at FLC [82]. A SET class H3K4 methylase ARABIDOPSIS TRITHORAX-RELATED 7 (ATXR7) binds directly to *MIR156A* to regulate the levels of H3K4me3 but not H3K27me3 [76]. The SWI2/SNF2 Related 1 (SWR1) complex mediates the exchange of histone H2A to histone H2A.Z, which favors H3K4me3 at *MIR156A/MIR156C* and prevents precocious vegetative phase change transition [76] (Figure 3a,c). ATXR7 functions synergistically with SWR1 complex components ACTIN-RELATED PROTEIN 6 (ARP6) and SERRATED LEAVES AND EARLY FLOWERING (SEF) in regulating vegetative phase change. Western analysis on the abundance of H3K4me3 and H3K27me3 in the shoots revealed that ARP6 and SEF do not affect the levels of these methylation marks globally [76]. 

Since PRC1 and PRC2 are both involved in regulating H3K27me3 levels during embryo-to-seedling transition, and chromatins of *MIR156A/MIR156C* are marked by H3K27me3, it is likely that PRC1 and PRC2 are also involved in regulating juvenile-to-adult phase transition. AtBMI1 and VAL1/2 have been shown to be involved in regulating vegetative phase change by modulating the levels of H2Aub and H3K27me3 at *MIR156A/MIR156C* [78]. VAL1/2 bind to the RY motif of *MIR156A/C* chromatin constitutively and they modulate the abundance of H2AK121ub at *MIR156A/C* and H3K27me3 at *MIR156C*, suggesting that VAL1/2 are required for the recruitment of both PRC1 and PRC2 [83]. Consistent with this, VAL1/2 physically interact with MSI1 [83,84]. VAL1/2 also regulate the activities of SPL9 independently of miR156, indicating that VAL1/2 function in both miR156-dependent and miR156-independent pathways [83]. SPL9 is highly occupied by H2AK121ub with little H3K27me3, suggesting that PRC1, not PRC2 is involved in regulating SPLs [83]. Consistent with this, PRC1 components AtRING1A/B have been shown to mediate H2Aub1 levels at *SPL3*, *SPL9* and *SPL10* [79] (Figure 3b,d). miR156 is encoded by eight genes and miR157 is encoded by four genes, and 10 of these 12 genes are marked by H3K27me3 [7]. The exact PRC1 and PRC2 components involved in depositing H3K27me3 at these genes are not clear and it is unknown if these miR156 and miR157 genes are regulated by similar or different mechanisms. 

### 3.3. Vegetative-to-Reproductive Phase Transition Mediated by PRC2 and PRC1

The vegetative-to-reproductive transition (floral induction) in *Arabidopsis* is mediated by both endogenous factors like age and the plant hormone gibberellins, as well as environmental factors such as temperature and day length [85]. These signals are integrated to form a regulatory network to control flowering time. Among those factors, cold winter (vernalization pathway) primarily involves FLC, which acts as a central floral repressor [85]. Silencing of *FLC* is maintained by the VRN2-PRC2 complex, which has been described in several reviews and will not be discussed in detail here [1,85,86,87]. 

**Figure 4:**
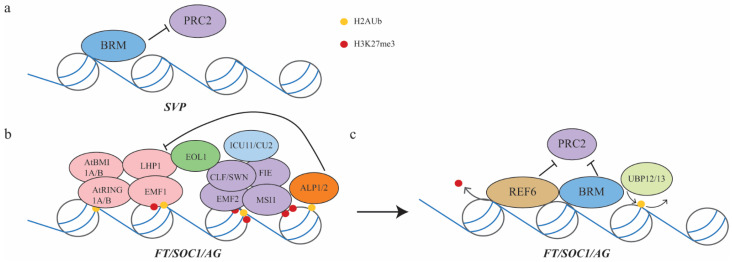
Model of PRC1 and PRC2 action during vegetative-to-reproductive phase transition. (**a**) BRM binds to the flowering repressor *SVP* to prevent PRC2 binding and maintain *SVP* activity during vegetative development. (**b**) Flowering activators *FT/SOC1/AG* are repressed after germination by the activities of both PRC1 and PRC2 to prevent precocious flowering and allow proper vegetative development. Both PRC1 and PRC2 bind to them. EOL1 and ICU11/CU2 act as PRC1 or PRC2 accessory proteins to assist with silencing. (**c**) Upon floral induction, UBP12/13 remove H2Aub, while REF6 removes H3K27me3 and recruits BRM at *FT/SOC1/AG* to prevent further PRC2 binding and allow activation of *FT/SOC1/AG*.

Mutations in *EMF1* and *EMF2* result in precocious upregulation of floral activators such as *FLOWERING LOCUS T* (*FT*), *SUPPRESSOR OF OVEREXPRESSION OF CO1* (*SOC1*) and *AGAMOUS* (*AG*), and flowering right after germination without developing any true leaves [15,20,32,88,89], suggesting that PRC1 and PRC2 are required to suppress precocious early flowering (Figure 4b). Consistent with this, mutations in *CLF* resulted in early flowering, likely caused by reduced levels of H3K27me3 at *SOC1*, *FT*, *AG*, *AGAMOUS*-like *17* (*AGL17*), *AGL19*, *AGL24*, and *AGL71*, and consequently upregulation of these genes in the shoot [90,91,92,93]. On the other hand, the floral repressor *SVP* is devoid of H3K27me3 to prevent early flowering [81]. BRM binds to *SVP* directly to prevent the binding of PRC2 and deposition of H3K27me3 at *SVP* to prevent early flowering [81] (Figure 4a). Upon floral induction, the floral activators are upregulated for reproductive success. The H3K27me3 levels in *FT* and *SOC1* are therefore reduced by histone demethylase RELATIVE OF EARLY FLOWERING 6 (REF6) (Figure 4b). *REF6* encodes a Jumonji domain-containing protein and loss of function *REF6* is late flowering while overexpression of *REF6* resulted in early flowering [94]. The zinc finger domain of REF6 is essential for its binding to the CTCTGYTY motif (where Y represents T or C) of its targets [95]. REF6 also physically interacts with BRM, and they colocalize at 1276 targets in *Arabidopsis* to prevent further PRC2 binding [95,96] (Figure 4c). 

Several accessory proteins have been discovered to aid PRC2 in floral induction. *lhp1* and *clf* could be partially rescued by *antagonist of like heterochromatin 1* (*alp1*), and molecular and genetic analysis showed that ALP1/2 are PRC2 antagonistic accessory proteins as they competitively bind to MSI1, thus releasing LHP1 from binding to PRC2 and prevent stable silencing [97]. A mutation in *ENHANCER OF LPH1* (*EOL1*), the Yeast Chromosome transmission fidelity 4 (Ctf4)-like gene in *Arabidopsis*, enhances the *lhp1* and *clf* early flowering phenotype [98]. EOL1 physically interacts with LHP1 and CLF, and together they mediate the deposition of H3K27me3, suggesting that EOL1 assists with the activities of PRC1 and PRC2 [98]. *INCURVATA11* (*ICU11*), encoding a 2-oxoglutarate-dependent dioxygenase, has been reported to have histone demethylation activity in other organisms [99,100]. In *Arabidopsis*, the *icu11* mutant resembles *clf* leaves by having upward curly leaves. Mutations in *icu11* and its close paralog *cupuliformis2* (*cp2*)result in flowering without any vegetative growth, mimicking *emf1* or *emf2* mutants [100]. ICU11 robustly interacts with PRC2 core components and binds to the nucleation region of *FLC* to mediate the demethylation of H3K36me3 (an active marker at *FLC*) (Figure 4b), suggesting that it controls the switch between H3K36me3 and H3K27me3 at *FLC* [99]. Together, ALP1, EOL1, ICU11, and CP2 are accessory proteins of PRC2.

### 3.4. How Is PRC2 Recruited and How Is H3K27me3 Spread?

Accurate temporal recruitment of PRC2 to its targets is essential for its activity. Both cis- and trans-acting elements have been investigated to reveal the recruitment mechanism of PRC2. The cis elements here refer to the DNA sequence elements that PRC2 binds to and are termed Polycomb Response Elements (PREs). Genome wide analysis of PRC2 binding by FIE-HA ChIP seq revealed that PREs consist of a telobox binding site, a zinc finger protein binding site, and a GAGA factor binding site [18]. Computational analysis on the PRC2 binding sites revealed that the CTCC, CCG, G-box, GA-repeat, AC-rich and telo-box motifs are the PREs in *Arabidopsis* [19]. Genome-wide analysis of CLF-GFP and SWN-GFP binding by ChIP seq confirmed that *GAGA*-like motif and *telo*-box-like motif are PREs in *Arabidopsis* [93]. The *telo*-box motif has been shown to be recognized by Telomere-Repeat Factors (TRBs), which bring CLF and SWN to targets by physical interaction with CLF and SWN [101]. In addition to TRBs, the C2H2 zinc-finger (ZnF) family, the plant-specific APETALA2-like family, and the plant-specific BASIC PENTACYSTEINE (BPC) family of transcription factors could also be PRC2 recruitment factors, as they bind to PREs in yeast-one-hybrid assays and are colocalized with FIE and H3K27me3 [19]. 

During embryo-to-seedling transition, the transcription factors VAL1/2 mediate the recruitment of PRC1, which further recruits PRC2 to methylate H3K27 [11,35]. VAL1/2 are also recruited to the nucleation region of *FLC*, which further recruit histone deacetylase HDA19, PRC2, and PRC1 to mediate H3K27me3 accumulation at *FLC* [84]. H3K27me3 levels are gradually increased at the nucleation region during vernalization by the activities of VRN2-PRC2. H3K27me3 spreads from the nucleation region toward the 3′ end after the plant is returned to warm temperature, but the binding of VRN2 at the *FLC* chromatin decreases when H3K27me3 spreads [102,103,104]. Recent studies show that the spreading of H3K27me3 across the *FLC* gene body in warm conditions is mediated by CLF and LHP1, which maintains the long-term stable silencing of *FLC* [105]. The overall trend of H3K27me3 spreading has been documented across the genome, but the details of how H3K27me3 is spread remain largely unknown [93].

## 4. PRC1 and PRC2 in Plants Other Than *Arabidopsis*

PRC1 and PRC2 are also found in plants other than *Arabidopsis*. Evolutionary studies on the PRC1 subunits (LHP1, Ring1A/1B, BMI1A/1B/1C, EMF1, and VRN1) show that the PRC1 core subunits are conserved in angiosperms [34], indicating that PRC1 may play similar important regulatory roles in flowering plants. Homologous genes of *LHP1*, *BMI1A/1B/1C*, *RING1A/1B*, and *EMF1* have been discovered in the allohexaploid bread wheat (*Triticum aestivum*) [106], providing vital tools for studying and manipulating PRC1 in this plant. *RING1*, *BMI1* and *LHP1* are also present in early plant homologs, and have been found in mosses, lycophytes and ferns [34,107]. Specific analysis of PRC1 components in *Physcomitrella patens* (a moss) showed that the *Physcomitrella* RING1A/B (Pp RING1A/B) physically interacts with PpBMI1A/B and PpLHP1 in the nucleus, similar interactions observed in *Arabidopsis* [34,107]. Interestingly, it was also reported that PpCLF associates with PpRING1A/B, indicating direct interaction between PRC1 and PRC2 in moss [108]. Homologous genes of PRC2 were discovered in single-celled plants as well as flowering plants. In the single-celled algae *Chlamydomonas reinhardtii,* an Enhancer of zeste homolog (EZH) has been identified, indicating the possible early development of PRC2 in eukaryotes [109]. In *Physcomitrella patens,* PpFIE and PpCLF were shown to promote cell differentiation of the gametophyte (haploid) to sporophyte (diploid) transition [110,111]. Additionally, the *Arabidopsis FIE* could partially rescue the *Ppfie* mutant, and *PpFIE* could partially complement the *Arabidopsis fie* mutant, indicating overlapping and distinct functions of FIE in moss and flowering plants [110]. Given the conserved regulatory roles of PRC2 in flowering plants, the discovery of the PRC2 homologs could have great potential in improving agricultural traits in crops. In rice (*Oryza sativa*), two homologs of E(z)-like genes, (*OsEZ1* and *OsCLF*), two homologs of Su(z)12 (*OsEMF2a* and *OsEMF2b*), and two homologs of ESC-like genes (*OsFIE1* and *OsFIE2*) have been discovered [112]. OsFIE2 is responsible for the accumulation of H3K27me3 in rice, but the *osfie2* mutant does not show autonomous endosperm development like the *fie* mutants in *Arabidopsis*, suggesting different roles of FIE in rice and *Arabidopsis* [113]. Alternatively, one T-DNA insertion line of *osemf2b* showed defects in flowering time (early flowering) and floral organ development, indicating a conserved role of EMF2 in rice and *Arabidopsis* [112]. Accessory homeodomain proteins like OsVIN3-LIKE 2 (OsVIL2) have also been found to directly interact with OsEMF2b to modulate the abundance of H3K27me3 at *O. sativa LEAFY COTYLEDON 2 and FUSCA 3-LIKE 1* to regulate flowering in rice [114]. The homologs of PRC2 in maize (*Zea Mays*) have been duplicated many times, with five homologs of *MSI1*, three homologs of E(z), two of ESC and two of Su(z)12 identified in maize [115]. In barley (*Hordeum vulgare)*, *HvSu(z)12a*/b/c, *HvE(Z)*, and *HvFIE* have been identified as Suz12 homologs, with H*vSu(z)12b* being involved in all tissues, *HvSu(z)12c* was most abundant in young shoots, and *HvSu(z)12a* being nearly undetectable [116]. 

## 5. Future Perspectives

PRC1 and PRC2 control plant developmental transitions at different stages. Though there have been great advances in how PRC2 is recruited, spread and maintained in *FLC*, it is still largely unknown how PRC2 operates at other target genes such as *LEC1*, *LEC2*, *ABI3* and *MIR156A/C* during embryo-to-seedling transition or juvenile-to-adult vegetative phase transition. There are a large number of genes bound by PRC1 (EMF1) that are devoid of H3K27me3, suggesting that PRC1 also functions independently of PRC2 [88]. Dissecting the components of PRC1 and PRC2 in each phase transitions and determining how they act is essential for us to the understanding of how the PRC1 and PRC2 complexes function during phase transitions.

Several routes of investigation are being pursued to deepen our knowledge of these mechanisms. Recent studies have continued to examine the role of transcription factors in the recruitment of PRC1 and PRC2. VAL1/2 recruit both PRC1 and PRC2 to the targets and increase H2Aub and H3K27me3 occupancy while simultaneously decreasing the levels of H3ac [117], suggesting the involvement of histone deacetylase (HDAC) with PRC1 or PRC2. VAL1 directly interacts with HDA9 or interacts with HDA19 via SAP18 in regulating *FLC* [84,118], confirming the involvement of HDAC in PRC1 and PRC2 activities. VAL1/2 are also involved in the temporal expression of *MIR156A/C* in vegetative phase change [83], but which HDAC are involved and how they act to regulate the PRC2 activities at *MIR156A/C* remains to be elucidated. Besides VAL1/2, other transcription factors with EAR domain could recruit PRC2 and SIN3-histone deacetylase complexes. The SIN3-HDAC complex in *Arabidopsis* consists of a SIN3-like protein, a HDAC, and MSI1 [119]. Together, this suggests that transcription factors and HDAC complexes could work in concert with PRC2 to mediate long-term gene silencing. However, how these factors are dynamically involved during each of the developmental phase transitions remains to be discovered.

Hormones are also beginning to be examined in relation to the epigenetic pathways. Hormones are involved in seed germination and floral induction [120,121]; however, it is as yet unclear whether hormones crosstalk to epigenetic factors in mediating developmental phase transitions. It has been found that abscisic acid (ABA) triggers the demethylation of H3K27me3 at SNF1-RELATED PROTEIN KINASES 2.8, an ABA-activated protein kinase, via Jumonji domain protein JMJ30 and JMJ32 thus initiating the study on crosstalk between hormones and epigenetic silencing of target genes [122], but such crosstalk in the developmental stages outside of the stress response has yet to be discovered.

Finally, studies have been analyzing the relationship between PRC1 and PRC2 and the three-dimensional structure of chromatin. Hi-C analysis in WT and *lhp1* indicated that LHP1, as part of the PRC1 complex, interacts with both intra- and inter-chromosomal DNA in a 3-D manner by forming loops in the DNA to bring specific genes closer to different regions for regulation [123]. This chromatin structure regulation opens yet another opportunity to find PRC1 and PRC2 interactions with phase transitions. Taken together, there are many avenues of research to be explored in the future to fully understand the complexity of polycomb repressing complexes and their regulation during phase transitions in plants.

## Figures and Tables

**Table 1:** Core subunits of PRC1 and PRC2 in different organisms.

	Mammals	*Drosophila*	*Arabidopsis*	Characteristic Domain	Activities
PRC1	RING1A/ RING1B	dRing/ Sce	AtRING1A/ 1B	RING finger domain and Ring-finger and WD-40 Associated Ubiquitin-Like (RAWUL) domain	E3 ubiquitin ligase activity for H2A
	PCGF1-6	Psc	AtBMI1A/ 1B/1C	RING finger domain and RAWUL domain	Co-factors for E3 ubiquitin ligase and compact nucleosomes
	CBX2/4/6/7/8	Pc	LHP1(TFL2)/ VRN1	Chromodomain and chromo shadow domain	Recognizes and binds to H3K27me3
	PHC1/ PHC2/ PHC3	Ph	UNKNOWN	Sterile Alpha Motif (SAM) domain and Zinc Finger domain	Mediates monoubiquitination of histone H2A
PRC2	EZH1/2	E(z)	CLF/SWN/ MEA	SET domain	H3K27 methyltransferase
	SUZ12	Su(z)12	EMF2/VRN2 FIS2	Zinc Finger	Assists with E(z) catalytic activity
	EED	Esc	FIE	WD-40 repeat domain	Assists with E(z) catalytic activity
	RBAP48/46	P55/Nurf55	MSI1-5	WD-40 repeat domain	Histone binding
